# Gold nanoparticles supported on magnesium oxide for CO oxidation

**DOI:** 10.1186/1556-276X-6-435

**Published:** 2011-06-22

**Authors:** Sónia AC Carabineiro, Nina Bogdanchikova, Alexey Pestryakov, Pedro B Tavares, Lisete SG Fernandes, José L Figueiredo

**Affiliations:** 1 Laboratório de Catálise e Materiais, Departamento de Engenharia Química, Faculdade de Engenharia, Universidade do Porto, 4200-465 Porto, Portugal; 2 Universidad Nacional Autónoma de México, Centro de Nanociencias y Nanotecnología, Carretera Tijuana-Ensenada, 22800 Ensenada, Baja California, Mexico; 3 Tomsk Polytechnic University, 30, Lenin Avenue, Tomsk 634050, Russia; 4 Universidade de Trás-os-Montes e Alto Douro, CQVR Centro de Química-Vila Real, Departamento de Química, 5001-911 Vila Real, Portugal

## Abstract

Au was loaded (1 wt%) on a commercial MgO support by three different methods: double impregnation, liquid-phase reductive deposition and ultrasonication. Samples were characterised by adsorption of N_2 _at -96°C, temperature-programmed reduction, high-resolution transmission electron microscopy, energy-dispersive X-ray spectroscopy and X-ray diffraction. Upon loading with Au, MgO changed into Mg(OH)_2 _(the hydroxide was most likely formed by reaction with water, in which the gold precursor was dissolved). The size range for gold nanoparticles was 2-12 nm for the DIM method and 3-15 nm for LPRD and US. The average size of gold particles was 5.4 nm for DIM and larger than 6.5 for the other methods. CO oxidation was used as a test reaction to compare the catalytic activity. The best results were obtained with the DIM method, followed by LPRD and US. This can be explained in terms of the nanoparticle size, well known to determine the catalytic activity of gold catalysts.

## Introduction

It is well known from the literature that for gold to be active as a catalyst, a careful preparation is needed to obtain nanoparticles well dispersed on the support [1-4]. Compared with other supports, MgO is considered as "inactive" [5-8] since it is basically an irreducible oxide, such as Al_2_O_3_. These materials have low ability to adsorb or store oxygen at low temperatures [5].

However, Margitfalvi et al. [9] prepared Au/MgO catalysts with high activity for low temperature CO oxidation. The activity of these catalysts was further increased by modification with ascorbic acid in a relatively narrow concentration range. These authors suggested that the addition of ascorbic acid slightly changes the ionic/metallic gold ratio and suppresses formation of carbonate, which is responsible for deactivation [9]. Gates and co-workers [10,11] also managed to produce a Au/MgO catalyst that was active for CO oxidation at 30°C by bringing Au(CH_3_)_2_(acac) (acac is acetylacetonate) in contact with partially dehydroxylated MgO and by treatment in flowing helium at 473 K, during which the original mononuclear Au(III) species decomposed, gold being reduced and aggregated. The catalyst underwent rapid deactivation due to the formation of carbonate-like species on the support and on gold, but could be reactivated by treatment in flowing helium, which led to the removal of the carbonate-like species [10].

Heinz et al. [12] showed that small clusters of gold (Au_20 _and Au_8_) are active towards CO oxidation. In fact, for Au_8 _clusters, it was found that the oxidation of CO at -33°C is activated after deposition on defect sites of the MgO support [13,14]. Guzman and Gates [15-17] showed, by X-ray absorption spectroscopy, the presence of both cationic and reduced gold in MgO-supported gold clusters during CO oxidation. Molina and Hammer [18] showed by DFT calculations that O_2 _can bind simultaneously to both metal centres (Au and Mg) with CO bonded to another nearby Au centre. Broqvist et al. [19] proved also by DFT calculations that Cl was a poison for Au/MgO catalysts in CO oxidation, while Na was a promotor. Goodman and co-workers [20] showed a direct correlation between the concentration of F-centre surface defects in the MgO support and the catalytic activity for CO oxidation of the subsequently deposited Au, implying a critical role of surface F-centres in the activation of Au in Au/MgO catalysts.

Grisel and Nieuwenhuys [21] found that Au/MgO catalysts supported on alumina were extremely active, achieving 50% CO conversion at room temperature and full conversion at approximately 250°C. It is, however, worth to note that those materials had 5% Au loading, while 1% Au was used in this study. Moreover, these authors used 2% CO in the gas feed for the CO oxidation experiments, while we used 5% CO. Szabó et al. [22-24] also reported that Au/Al_2_O_3 _catalysts modified by MgO exhibited high activity in the sub-ambient and ambient temperature ranges for CO oxidation.

Co-precipitation (CP) [1-5,25-31] and deposition-precipitation (DP) [1-4,6,21,22,29,31] are the most common methods to prepare oxide-supported gold catalysts. In this study, less usual Au loading methods were used, such as double impregnation (DIM) [32] and liquid phase reductive deposition (LPRD) [33], to prepare Au nanoparticles. To the best of our knowledge, the only reports on the use of DIM is the work of Bowker et al. [32] dealing with TiO_2 _samples and our previous work on CeO_2 _[34,35] and ZnO [36] catalysts. This method represents an environmentally and economically more favourable route to the preparation of high activity gold catalysts, in comparison to the traditional deposition-precipitation (DP) method [32]. As far as we know, LPRD has only been used by Sunagawa et al. [33] to prepare Pt and Au catalysts on Fe_2_O_3_, FeOOH, ZrO_2 _and TiO_2 _supports, and also by us for CeO_2 _[37] and TiO_2 _[38]. US was only used by our group to prepare very active Au/ZnO catalysts [36].

The aim of this study is to compare the activity for CO oxidation of Au/MgO catalysts prepared by these unusual methods. This is a simple model reaction to evaluate gold catalysts that has many potential applications, namely in CO removal from H_2 _streams for fuel cells and gas sensing [1-4,34,36,37].

## Experimental

Commercial MgO (p.a., Merck) was used as received and after a treatment at 400°C, in N_2_, for 2 h.

### Preparation of Au catalysts

Au was loaded on the MgO support by the double impregnation method (DIM) [32], liquid phase reductive deposition (LPRD) [33] and ultrasonication (US) [36]. Briefly, the first method (DIM) consists in impregnating the support with an aqueous solution of the gold precursor (HAuCl_4_) and then with a solution of Na_2_CO_3 _that precipitates gold hydroxide within the pores of the catalyst [32,34-36]. The second procedure (LPRD) consists of mixing a solution of HAuCl_4 _with a solution of NaOH (with a ratio of 1:4 in weight) that hydroxylates the Au^3+ ^ions, before the support is added to the solution [33,37,38]. Au^3+ ^ions are reduced to metallic Au^0 ^by electron transfer from coordinated OH^- ^ions on the surfaces of support particles through their catalytic action [33]. US consists in dissolving the Au precursor in water and methanol, and sonicating for 8 h, reducing gold [36]. In all these methods, a washing procedure is carried out to eliminate residual chloride, which is well known to cause sinterization of Au nanoparticles, turning them inactive [1-4,37]. Further details can be found elsewhere [34-38].

### Characterization techniques

The materials were analysed by adsorption of N_2 _at -196°C in a Quantachrom NOVA 4200e apparatus.

Temperature-programmed reduction (TPR) experiments were performed in a fully automated AMI-200 Catalyst Characterization Instrument (Altamira Instruments, Pittsburgh, PA, USA), equipped with a quadrupole mass spectrometer (Dymaxion 200 amu, Ametek). Further details can be found elsewhere [34-38].

High-resolution transmission electron microscopy (HRTEM) measurements were performed with a JEOL 2010 microscope with a point-to-point resolution better than 0.19 nm. The sample was mounted on a carbon polymer-supported copper micro-grid. A few droplets of a suspension of the ground catalyst in isopropyl alcohol were placed on the grid, followed by drying at ambient conditions. The average gold particles and the particle size distribution were determined from a count of at least 250-300 particles. Semi-quantitative estimation of gold loading was performed by energy-dispersive X-ray spectroscopy (EDXS).

X-ray diffraction (XRD) analysis was carried out in a PAN'alytical X'Pert MPD equipped with a X'Celerator detector and secondary monochromator. Rietveld refinement with PowderCell software [39] was used to identify the crystallographic phases present and to calculate the crystallite size from the XRD diffraction patterns. Further details can be found elsewhere [34-38].

### Catalytic tests

Catalytic activity measurements for CO oxidation were performed using a continuous-flow reactor. The catalyst sample (0.2 g) was placed on a quartz wool plug in a 45-cm long silica tube with 2.7 cm i.d., inserted into a vertical furnace equipped with a temperature controller. Feed gas (5% CO, 10% O_2 _in He) was passed through the catalytic bed at a total flow rate of 50 ml · min^-1 ^(in contrast with most literature studies that use 1% CO or less [1-4,31]). The composition of the outgoing gas stream was determined using a gas chromatograph equipped with a capillary column (Carboxen 1010 Plot, Supelco) and a thermal conductivity detector. Further details can be found elsewhere [34-38].

## Results and discussion

### Characterization of samples

#### BET surface area

The BET surface area obtained for the MgO sample by N_2 _adsorption at -196°C was 32 m^2 ^· g^-1^. This value is smaller than those reported in the literature [9,23]. Both the thermal treatment of the support at 400°C and/or addition of gold by any of the methods described did not produce significant changes in the BET surface area.

#### XRD

**Figure 1:**
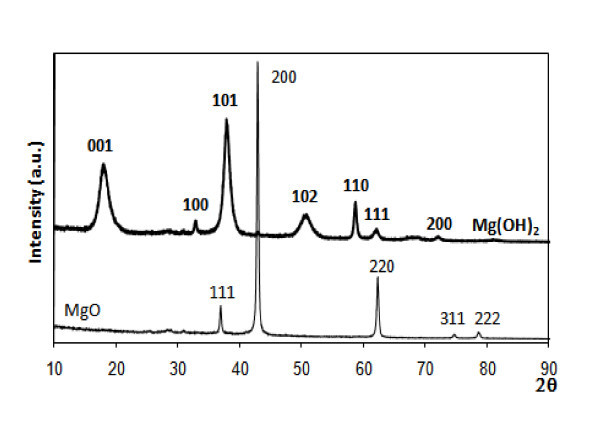
**X-ray diffraction spectra of commercial MgO, pure (thin line) and loaded with 1% Au wt (thicker line) by DIM, with phases and respective crystal planes (Miller indexes) identified**.

Figure 1 shows the XRD spectra of the oxide supports alone, and loaded with 1 wt% Au by DIM. The identified phase for the unloaded material is the respective oxide (cubic, Fm-3m, 01-078-0430), with a crystallite size of 42 nm; however, when gold is loaded, a new Mg(OH)_2 _phase (hexagonal, P-3m1, 01-076-0667) was formed (Figure 1). 99% of this hydroxide phase was detected along with 1% MgO. It was not possible to calculate the particle size of the Mg(OH)_2 _phase due to interstratification of hydrated phases, as also found by other authors [40], which makes it very difficult to simulate the spectra, so the results obtained (in this case approximately 25 nm) are not reliable. The hydroxide is most likely formed by reaction with water, in which the gold precursor is dissolved (MgO + H_2_O → Mg(OH)_2_). Similar results were obtained for the other loading methods.

The Au particle size could not be determined for any of the gold-loaded samples through XRD analysis, since the characteristic XRD reflection was absent in these materials. This can be due to the low loading (1 wt%) and small size of Au particles present in these catalysts, as it will be seen by HRTEM.

#### HRTEM

**Figure 2:**
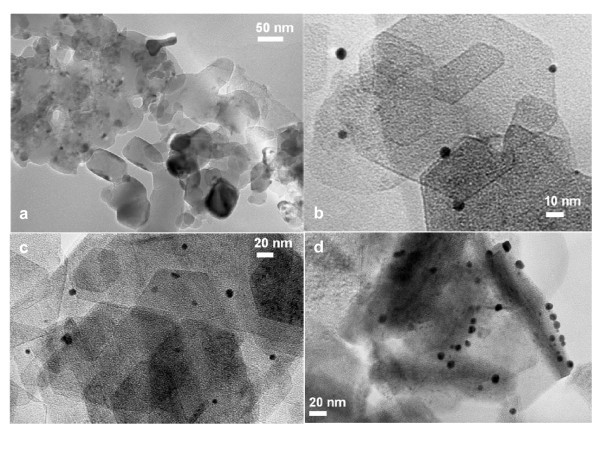
**HRTEM images of the commercial MgO, pure (a) and loaded with 1% Au wt by DIM (b), LPRD (c) and US (d)**.

**Figure 3:**
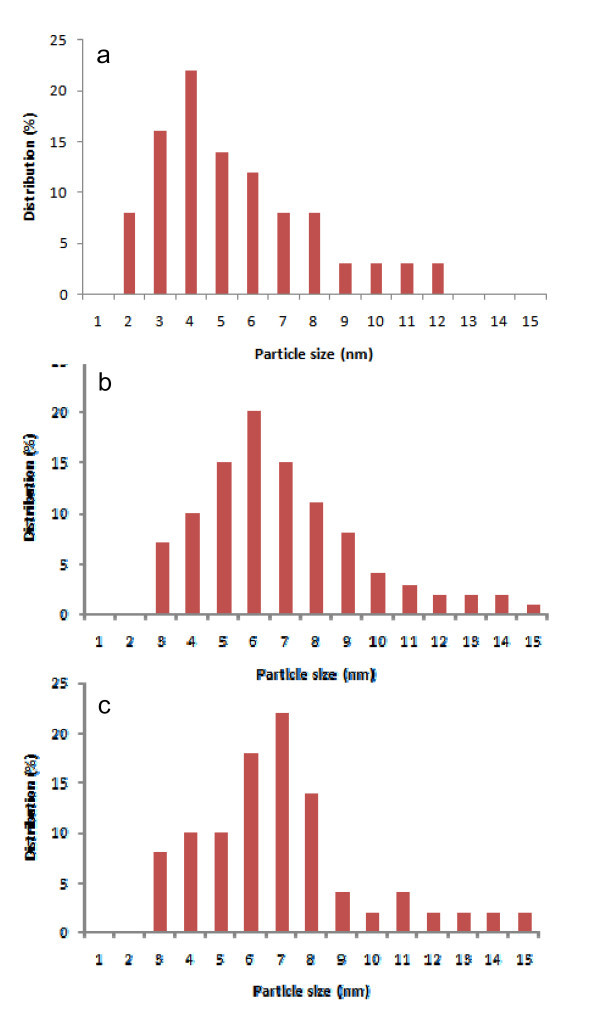
**Size distribution histograms of Au nanoparticles on MgO, prepared by DIM (b), LPRD (c) and US (d), with respective average sizes**.

Figure 2a shows a HRTEM image of the MgO support which is quite different from what is observed in Figure 2b, c, d (MgO with Au loaded by DIM, LPRD and US, respectively), as the support changes from large crystals (Figure 2a) into a different structure (Figure 2b, c, d). Figure 3 shows the Au nanoparticle size distributions on MgO, prepared by the different methods. Gold particles are also observed with sizes ranging from 2 to 12 nm for DIM (Figures 2b, 3a). Other methods showed larger gold nanoparticle sizes between 3 and 15 nm (Figures 2c, 3b for LPRD and Figures 2d, 3c for US). The average size of gold particles is 5.4 nm for DIM and 6.6 nm for LPRD. US showed a slightly larger average gold size (6.7 nm), however the particles were closer to each other (Figure 2d).

Gold nanoparticles of 6 nm were reported in literature for Au/MgO catalysts prepared by CP [5]. Smaller values of approximately 4 nm were however obtained by CP and DP on Mg(OH)_2 _[5,41,42]. Sizes of approximately 4 nm were also obtained for Au on MgO prepared from a gold complex [20]. Au nanoparticles smaller than 5 nm were obtained on MgO modified with ascorbic acid [9,23]. Other techniques like impregnation produced gold particles of 8 nm on MgO [43]. Values of approximately 9 nm were obtained for gold on MgO with cube morphology [8]. Gold deposited on MgO/alumina yielded particles ranging from 2.7 to 4.6 nm [21,22,24,44].

#### EDXS

Semi-quantitative estimation of gold loading was performed by EDXS, approximately 0.9% being found for all samples.

#### TPR

**Figure 4:**
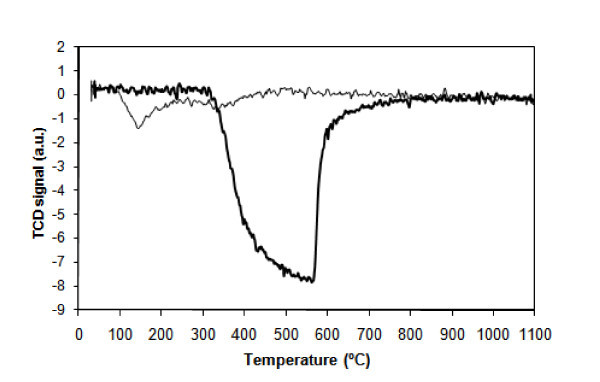
**H_2_-TPR profiles of the commercial MgO, pure (thin line) and loaded with 1% Au wt (thicker line) by DIM**.

TPR results are shown in Figure 4 for the pure MgO and MgO loaded with gold by DIM. It can be seen that pure MgO does not show any significant reduction peak in the studied range of temperatures (thin line), as expected from the literature [16,45]. When Au is loaded into MgO, as discussed above, the support is transformed into Mg(OH)_2_, most likely by reaction with water. As can be seen in Figure 4 (thick line), a large negative peak is observed on the TPR spectrum between approximately 300 and approximately 600°C. This means that hydrogen is not being consumed. However, water release was detected by mass spectrometry, most likely meaning that MgO is being formed (Mg(OH)_2 _→ MgO + H_2_O). In fact, a second TPR run produced a spectrum with no peaks, as for the oxide, as expected from the literature [16,45]. Similar results were obtained for samples loaded by the other methods.

#### Catalytic tests

**Figure 5:**
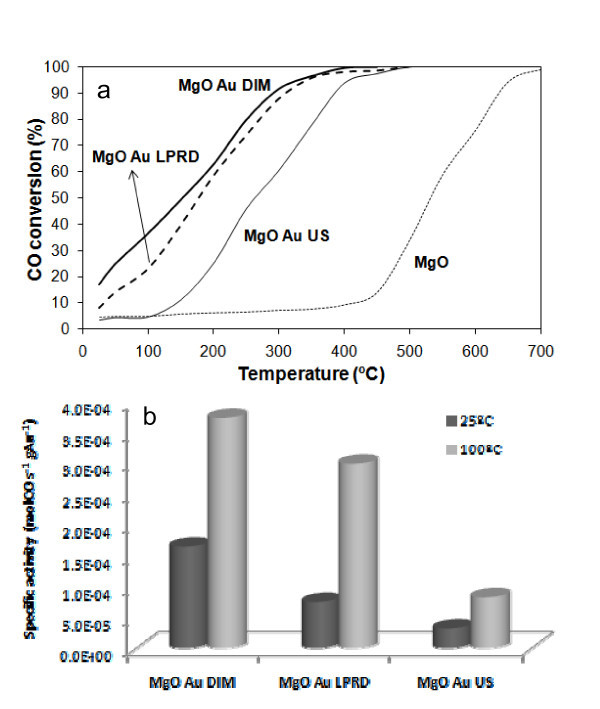
**CO conversion (%):** CO conversion (%) versus temperature for MgO supports alone and with Au loaded by different methods (a). Specific activities for the Au/MgO catalysts determined at 25 and at 100°C (b).

It was found that the activity for CO oxidation (with or without Au) of the heat-treated MgO did not improve when compared with the as-received oxide; therefore, only the results of the untreated samples are shown in Figure 5a. Loading MgO with Au causes total CO conversion to occur at much lower temperatures than with the support alone, as expected. DIM showed to be the best gold-loading method, followed by LPRD and US.

It can be argued that there are gold catalysts that achieve full CO conversions already at room temperature, but it has to be taken into account that most studies in literature use 1% CO or less [1-4] (while we used 5% of this gas). Also, the majority of authors use higher loadings of Au [1-4] (while we used 1 wt%). Nevertheless, it is possible to see, in our case, that CO conversion increases up to four times by addition of gold (for MgO with Au loaded by DIM), when compared to the unloaded samples.

Schubert et al. [5] reported activities of 13 × 10^-4 ^and 3.8 × 10^-4 ^mol_CO_g_Au_^-1 ^· s^-1 ^at 80°C for Au/Mg(OH)_2 _and Au/MgO catalysts, respectively, both prepared by CP, while Haruta's group obtained 1.2 × 10^-4 ^mol_CO_g_Au_^-1 ^· s^-1 ^at -70°C for a Au/Mg(OH)_2 _prepared by DP [46]. Our values for the DIM catalyst, ranging from 1.7 × 10^-4 ^to 3.8 × 10^-4 ^mol_CO_g_Au_^-1 ^· s^-1 ^at 25 and 100°C (Figure 5b), respectively, are similar to the literature value obtained with Au/MgO catalyst, but below the value obtained for the Au/Mg(OH)_2 _material [5]. Nevertheless, it was shown that the heat-treated samples (that have MgO instead of Mg(OH)_2_) have similar activity, meaning that the here reported DIM materials have similar catalytic activity to those reported in the literature, although with double Au content (1% Au, instead of 0.5% Au reported in [5]). LPRD and US showed smaller values.

## Conclusions

Au was loaded (1 wt%) on a commercial MgO support by three different methods: double impregnation (DIM), liquid-phase reductive deposition (LPRD) and ultrasonication (US). CO oxidation was used as a test reaction to compare the catalytic activity. The best results were obtained with the DIM method, which showed activities of 1.7 × 10^-4 ^to 3.8 × 10^-4 ^mol_CO_g_Au_^-1 · ^s^-1 ^at 25 and 100°C. This can be explained in terms of the nanoparticle size, well known to be related with the catalytic activity of gold catalysts. This sample had the narrowest size range (2-12 nm) and the lowest average size (5.4 nm). Samples prepared by other methods (LPRD and US) showed broader size ranges (3-12 nm) and larger average gold sizes (> 6.6 nm).

## Abbreviations

CP: co-precipitation; DP: deposition-precipitation; DIM: double impregnation; EDXS: energy-dispersive X-ray spectroscopy; HRTEM: high-resolution transmission electron microscopy; LPRD: liquid-phase reductive deposition; TPR: temperature-programmed reduction; US: ultrasonication; XRD: X-ray diffraction.

## Competing interests

The authors declare that they have no competing interests.

## Authors' contributions

SACC conceived the research work, prepared the catalysts, performed the activity tests, carried out the analysis and interpretation of the experimental results and drafted the manuscript. J.L. Figueiredo provided the means for the realization of this work and contributed to the writing. N.B. and A.P. performed the HRTEM experiments, while P.B.T. and L.S.G.F. carried out the XRD analyses. All authors read and approved the final manuscript.
